# Light intensity and spectral composition drive reproductive success in the marine benthic diatom *Seminavis robusta*

**DOI:** 10.1038/s41598-021-92838-0

**Published:** 2021-09-02

**Authors:** Gust Bilcke, Lore Van Craenenbroeck, Alexandre Castagna, Cristina Maria Osuna-Cruz, Klaas Vandepoele, Koen Sabbe, Lieven De Veylder, Wim Vyverman

**Affiliations:** 1 Protistology and Aquatic Ecology, Department of Biology, Ghent University, Krijgslaan 281 S8, 9000 Ghent, Belgium; 2 Department of Plant Biotechnology and Bioinformatics, Ghent University, Technologiepark 71, 9052 Ghent, Belgium; 3 VIB Center for Plant Systems Biology, Technologiepark 71, 9052 Ghent, Belgium; 4 Department of Applied Mathematics, Computer Science and Statistics, Ghent University, 9000 Ghent, Belgium; 5 Bioinformatics Institute Ghent, Ghent University, Technologiepark 71, 9052 Ghent, Belgium

**Keywords:** Gene expression analysis, Flow cytometry, Cellular microbiology, Environmental microbiology

## Abstract

The properties of incident light play a crucial role in the mating process of diatoms, a group of ecologically important microalgae. While species-specific requirements for light intensity and photoperiod have been observed in several diatom species, little is known about the light spectrum that allows sexual reproduction. Here, we study the effects of spectral properties and light intensity on the initiation and progression of sexual reproduction in the model benthic diatom *Seminavis robusta*. We found that distinct stages of the mating process have different requirements for light. Vigorous mating pair formation occurred under a broad range of light intensities, ranging from 10 to 81 µE m^−2^ s^−1^, while gametogenesis and subsequent stages were strongly affected by moderate light intensities of 27 µE m^−2^ s^−1^ and up. In addition, light of blue or blue–green wavelengths was required for the formation of mating pairs. Combining flow cytometric analysis with expression profiling of the diatom-specific cyclin *dsCyc2* suggests that progression through a blue light-dependent checkpoint in the G1 cell cycle phase is essential for induction of sexual reproduction. Taken together, we expand the current model of mating in benthic pennate diatoms, which relies on the interplay between light, cell cycle and sex pheromone signaling.

## Introduction

Diatoms are a highly diverse group of microalgae that occur in marine and freshwater as well as in terrestrial habitats and are responsible for about one fifth of global oxygen production^1,2^. Two groups of diatoms are distinguished based on the morphology of their unique silica cell wall called the frustule: the radially symmetric, generally homothallic centric diatoms and the bilateral pennate diatoms, which are mostly heterothallic^3–5^. Perhaps the most defining characteristic of diatoms compared to other microalgae is their cell size-dependent life cycle. With each mitotic division, the median population cell size decreases gradually until the species-specific sexual size threshold (SST) is reached, below which cells can engage in sexual reproduction^3^. In pennate diatoms, gametangia from compatible mating types (MT) assemble to form mating pairs, followed by gametogenesis, zygote and auxospore formation^3^. The elongated auxospore later hatches to release a large vegetative cell called the initial cell, thus restoring the maximal cell size^3^. In several pennate diatoms, a sex pheromone cascade guides partner recognition and mate finding^6–9^. Pheromone signaling is best understood in the benthic diatom *S. robusta,* where cells below the SST signal their presence to the compatible mating type using sex inducing pheromones (SIP), initially resulting in a cell cycle arrest in the G1 phase^10^. MT− then starts producing the attraction pheromone diproline, which causes the attraction of MT+ cells and formation of a mating pair^6,7^.

The marine underwater light field is highly complex, with steep spatial and temporal gradients in the scalar irradiance related to absorption by the water column, turbidity, weather conditions, time of day, and tidal cycles^11–13^. In addition, the spectral irradiance in the water column changes with increasing depth, depending on the interplay between the optical properties of the water column and the incident light field. Pure water strongly absorbs light in the red part of the spectrum, causing a dominance of blue light in clear oceans and lakes^11^. On the other hand, water containing abundant phytoplankton, detritus or coloured dissolved organic matter (CDOM) tends to absorb blue wavelengths, causing green or red light to dominate turbid habitats^14,15^. To perceive light, diatoms employ an extensive suite of molecular photoreceptors. Blue light can be perceived by members of the cryptochrome family^16^ and by aureochromes, light-driven transcription factors that are restricted to the stramenopiles^17,18^. Furthermore, diatom genomes encode heliorhodopsins, G-protein coupled receptor rhodopsin-like proteins and phytochromes, the latter specifically sensing red light^19–21^. Benthic diatoms have evolved multiple adaptations to cope with the dynamic light conditions and the strong attenuation of incident light by the sediment^22–24^. Many benthic diatoms exhibit maximal growth rates at relatively low photon flux densities (PFD, in this manuscript often simply referred to as “light intensity”) below 50 µE m^−2^ s^−1^, allowing them to thrive in shaded conditions^25–29^. In addition to physiological photoprotection, diatoms from the raphid pennate clade perform behavioural photoprotection by actively migrating through the sediment using their raphe, a longitudinal slit in the frustule equipped with an actin/myosin motility system^30,31^. Cells generally move upwards towards the sediment surface under moderate light intensities and migrate deeper into the sediment in darkness and under very high light intensities, resulting in synchronized rhythmic migration patterns over diurnal cycles^32–35^. Illumination experiments covering different wavelength ranges have shown that phototaxis and surface accumulation of motile diatoms is in most cases dependent on the presence of blue light^32,33,36,37^. *Stauroneis phoenicenteron*, on the other hand, is attracted to low intensity red light, suggesting that different benthic species employ different types of photoreceptors^24,38^.

Physiological evidence shows that the light regime can influence the progression of sex in both centric and pennate diatoms, even though the presence of a suitable partner has generally been considered the primary requirement for sexual reproduction in pennates^3^. Studies assessing the effect of light intensity and day length on reproductive success demonstrated large differences between species. While a shift to higher light intensities induced gametogenesis in several homothallic centric species^39–42^, dim light with an intensity below 50 µE m^−2^ s^−1^ was correlated with the highest level of auxospores in *Melosira nummeloides*, *Haslea ostrearia* and *Rhabdonema adriaticum*, and spermatogenesis in *Thalassiosira weissflogii* was almost completely abolished by light intensities above 100 µE m^−2^ s^−1^ ^43–45^. In terms of the photoperiod, species favored either a long day (e.g. 16:8 L:D), a short day (e.g. 8:16 L:D) or continuous light for sexual reproduction^41,43,45–49^. Notably, the proportion of sexually induced cells (gametangia) and initial cells in the pennate *Nitzschia lanceolata* was linearly dependent on the total amount of photosynthetic energy captured since the start of the photoperiod^28^. In contrast to light intensity and photoperiod, the spectral composition supporting sex has gathered little attention, although such data can provide crucial hints on which photoreceptors regulate the sexual light checkpoint. The formation of large cells in the centric *Chaetoceros didymus* was up to sevenfold higher under blue light compared to red light^50^, while auxospores were only observed after red light treatment in the pennate *H. ostrearia*^45^*,* suggesting that multiple independent light sensing mechanisms drive sexual reproduction in diatoms. Overall, physiological studies are fragmented and often fail to pinpoint the exact stages of the mating process that are susceptible to the light regime^3,43^.

The switch from the mitotic to the meiotic eukaryotic cell cycle almost universally occurs in the G1 phase, allowing the cell to integrate available signals before committing to the premeiotic S phase^51^. A similar situation has been observed in both centric and pennate diatoms. In case of *S. robusta* and *Pseudo-nitzschia multistriata,* this is accompanied by an intricate sex pheromone-induced G1 phase arrest*,* which postpones a potential transition to meiosis until the presence of a suitable partner in a mating pair is confirmed^7,9,10,52^. In addition to this sexually inducible region, the cell cycle of diatoms contains two light-dependent checkpoints, one in the G1 phase and a second at the G2/M boundary^53–55^. Outside these regions, the cell cycle can progress even in the absence of light^54^. Notable exceptions are the pennates *Phaeodactylum tricornutum* and *S. robusta*, where only G1 phase progression is light-dependent^56,57^. The molecular mechanism behind the light-dependent checkpoints of diatoms has to date only been assessed in *P. tricornutum*^58^*.* In this species, expression of the diatom-specific cyclin *dsCyc2* drives progression through the G1 checkpoint following light onset after a period of darkness^58^. Transcription of *dsCyc2* at dawn is regulated through the aureochrome photoreceptor *Aureo1a* in synergy with basic leucine zipper transcription factor *bZIP10*, and is therefore strictly blue-light dependent^58^. Recent transcriptomic evidence suggests that *Aureo1a* plays a much broader role as a master regulator of blue-light responsive genes, driving the expression of photoreceptors *CPF1* and *Aureo1c* as well as the transcription factor *bZIP11*^59^. Additionally, *Aureo1a* causes repression of photo-acclimation in high light^60^. Studying the relationship between light, cell cycle and spermatogenesis in the centric *T. weissflogii*, Armbrust et al*.* (1990) showed that the sexually inducible region is located downstream of the light-dependent checkpoint in the G1 phase^44^, but these findings have not yet been extended to other diatom species.

In this study, we investigate the sexual response of *S. robusta* to a range of light spectra and intensities. Standardized crossing protocols offered strict control over the mating process, allowing us to quantify different mating stages at predetermined time points. To characterize the genetic pathways behind light dependency, we assessed S phase progression with flow cytometry and quantified the expression of cell cycle genes using available RNA-seq datasets and by performing RT-qPCR on cultures treated with different spectra of light. Based on the combined information we introduce a hypothetical model for the complex regulation of sexual reproduction and vegetative growth in *S. robusta,* thus contributing to our understanding of the integration of light signals in the life of motile benthic diatoms.

## Results

### Gametogenesis is strongly affected by light intensity

**Figure 1:**
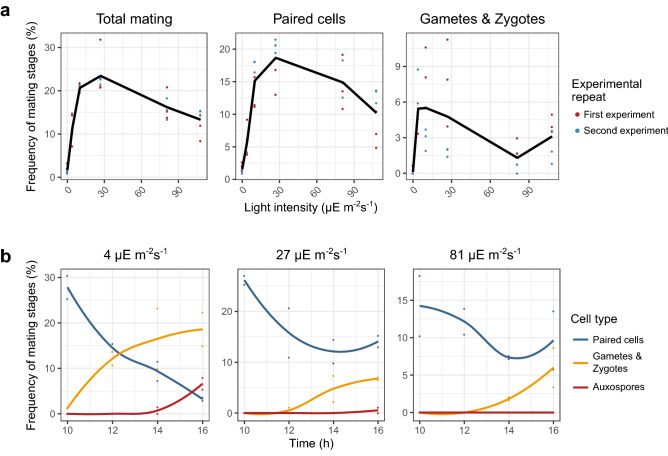
Influence of light intensity on sexual reproduction. (**a**) Influence of light intensity on the frequency of mating cell stages after 14 h of illumination. The experiment was performed twice, as indicated with colours. Dots represent frequency (in %) of sexual cells relative to all cell types (vegetative + sexual), while solid lines connect averages of both experiments. Confidence intervals for all pairwise combinations of intensities are visualized in Supplementary Fig. S1. (**b**) Time series showing the frequency of sexual cell stages over a period of 6 h after treatment with different light intensities (4, 27 and 81 µE m^−2^ s^−1^). Individual data points are represented by dots and the average percentage is connected by a Loess smoothed line. Different sexual cell stages are shown with different colours.

We assessed the reproductive success of *S. robusta* at different light intensities by exposing dark-synchronized crossed cultures (PONTON36 x PONTON34) to a broad range of intensities: 0, 4, 10, 27, 81 and 108 µE m^−2^ s^−1^, integrated over the full spectrum of the light source (“white” light). Sexual cell stages (paired cells, gametes & zygotes, auxospores and initial cells) were quantified after 14 h of illumination (Fig. 1a), which was selected as a reference time point because high levels of gametes and zygotes are consistently observed after such a period of time post-crossing. The experiment was performed twice to assess the consistency of the response to light intensity. The prevalence of sexual reproduction was severely reduced in the absence of light compared to all tested light intensities (*p* < 0.01, Supplementary Fig. S1). The persistent 2.0% and 1.1% paired cells that were counted in the dark likely represent the baseline level of cell pairs caused by random encounters between motile cells. The total reproductive frequency (summed proportion of all sexual cell types, i.e. paired cells, gametes, zygotes and auxospores) was highest at light intensities of 10 and 27 µE m^−2^ s^−1^*,* which produced significantly higher amounts of sexual cells compared to low (4 µE m^−2^ s^−1^) and high (108 µE m^−2^ s^−1^) intensities, and to 81 µE m^−2^ s^−1^ when the experiment was repeated (Fig. 1a, Supplementary Fig. S1). When focusing on the individual sexual cell stages, we observed that the overall response is dominated by the more abundant paired cells, and later stages show a distinct response. While the number of paired cells was maximal at intensities ranging from 10 to 81 µE m^−2^ s^−1^, gametogenesis and zygote formation peaked at intensities ranging from 4 to 27 µE m^−2^ s^−1^. The difference was even more pronounced when we repeated the experiment, as gametes and zygotes were significantly more numerous in very dim light (4 µE m^−2^ s^−1^) compared to all other intensities (Fig. 1a, Supplementary Fig. S1).

To better assess how light intensity affects the progression through each of the mating stages over time, a time-series experiment was set up consisting of crossed cultures subjected to three different light intensities: “low” (4 µE m^−2^ s^−1^), “moderate” (27 µE m^−2^ s^−1^) and “high” (81 µE m^−2^ s^−1^) (Fig. 1b, Supplementary Fig. S1). At the start of the time series (10 h), a high proportion (27.7%) of paired cells was present at low light intensities. As time progressed, the proportion of paired cells decreased (*p* < 0.001), alongside an increase of other cell stages, indicating progression through the mating process. This decrease in paired cells was significantly less steep in moderate and high intensities (*p* < 0.01 for both), suggesting that more intense light affects the transformation of paired cells into gametes/zygotes. Indeed, compared to low light, moderate to high light intensities displayed a significant delay in the production of gametes and zygotes, with the first gametes and zygotes being visible after 10 h, 12 h and 14 h in low, moderate and high light respectively (*p* < 0.05). Although a peak in the abundance of auxospores is expected later^61^, the increase of auxospores during the time series was already significantly lower when treated with moderate or high light intensities (*p* < 0.001). In fact, auxospores were never observed at high light intensity in the given timeframe.

### Blue light is essential to trigger the induction of sexual reproduction

**Figure 2:**
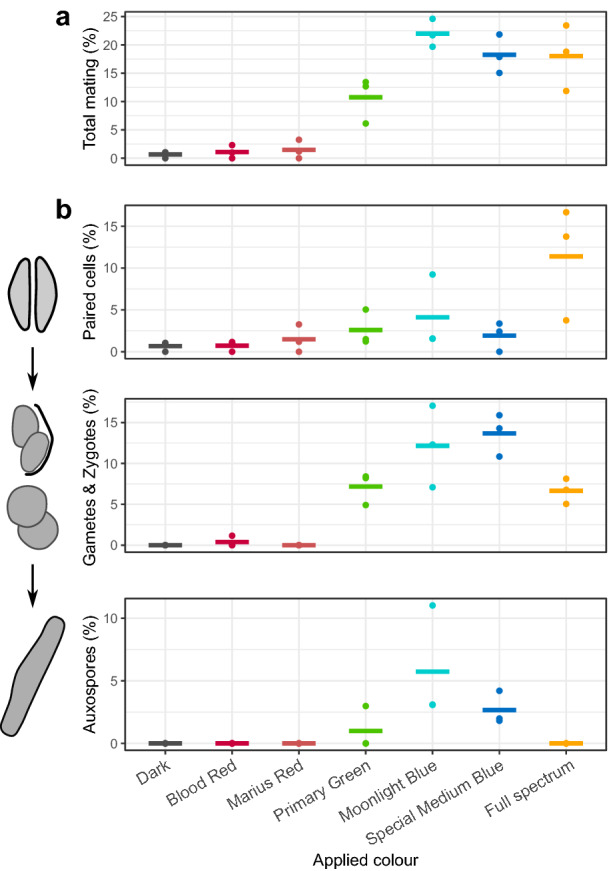
Influence of spectral composition on sexual reproduction. Frequency of mating cells in a crossed culture (PONTON36 x PONTON34) after 14 h of treatment in dark, full spectrum or different wavelength ranges transmitted by the colour filters. Points represent individual replicates, while horizontal lines show the average for each condition. Results are shown for the sum of all sexual cell types **(a)** and partitioned for each sexual stage **(b).** Confidence intervals for pairwise statistical tests are visualized in Supplementary Fig. S4.

The effect of spectral composition on sexual reproduction was assessed by exposing dark synchronized crossed cultures to different light sources using colour filters: one type of blue, one type of cyan, one type of green, two types of red as well as a full spectrum (“white”) and a dark control (Supplementary Fig. S2, S3). Since the colour filters did not transmit 100% of the light at the wavelength with maximal transmittance, the illumination was compensated to provide the same incident light intensity at this reference wavelength as for the treatment will full spectrum (Supplementary Table S1). In this way, cultures obtained approximately the same amount of light in the wavelength range of interest as they did in the control with the full spectrum (Supplementary Fig. S3). However, as a result, the total light intensity received by cultures is lower in filter-treated cultures compared to the full spectrum control (Supplementary Table S1). Sexual progression was assessed at the reference time point of 14 h. Corroborating previous findings, we observed a low percentage of paired cells (0.7%) and no other sexual stages in dark treated samples. Red light did not result in a distinguishable increase in total reproductive frequency compared to the dark (*p* > 0.9), illustrated by an absence of auxospores under these conditions. The total proportion of sexual cells for green (10.7%), cyan (22.0%), blue (18.3%) and full spectrum (18.0%) light was significantly higher compared to the dark or red light (*p* < 0.01) (Fig. 2a, Supplementary Fig. S4). Moreover, the cyan light treatment (“Moonlight Blue”) exhibited a significant increase compared to green light (*p* < 0.05). The spectrum below the Moonlight Blue filter covers both blue and green wavelengths, while Special Medium Blue is almost uniquely composed of blue wavelengths, and Primary Green of green wavelengths (Supplementary Fig. S3). The fact that the sexual response was highest in the filters containing a blue peak, suggests that mating is a predominantly blue-light response. When the data were analyzed by sexual cell stage, full spectrum, cyan and green light led to an increase of gametes and zygotes compared to the dark and red light (*p* < 0.001) (Fig. 2b, Supplementary Fig. S4). However, blue treatments had a significantly higher proportion of gametes and zygotes than the full spectrum treatment (*p* < 0.05) and no auxospores were identified under the full spectrum (Fig. 2b), likely as the result of the higher light intensity in the full spectrum control inhibiting gametogenesis (Supplementary Table S1). Since blue, cyan and full spectrum samples were treated with similar levels of blue light (Supplementary Fig. S3), the total PFD appears to be responsible for inhibition at the stage of gametogenesis, not only the photon flux in the blue spectrum.

### Indications for a blue light-dependent checkpoint during the G1 phase of the cell cycle in *S. robusta*

The results of our spectral range experiments showed that light requirements for sexual reproduction in *S. robusta* resemble those of the light-dependent G1 phase checkpoint in *P. tricornutum,* in particular the sensitivity towards blue wavelengths and low intensity light below 10 µE m^−2^ s^−1^ ^58^*.* Previous work has shown that the *S. robusta* sexually inducible cell cycle phase where SIP can trigger a cell cycle arrest is located in G1^7,10^. Hence, the lack of mating in the absence of blue light may reflect a failure to progress through a light-dependent checkpoint in the *S. robusta* G1 phase*.* We set out to verify (1) whether blue light is required for early cell cycle progression in *S. robusta,* and (2) if the known molecular components of this checkpoint in *P. tricornutum* are preserved in the *S. robusta* reference genome and their expression profile corroborates a light-dependent function.

**Figure 3:**
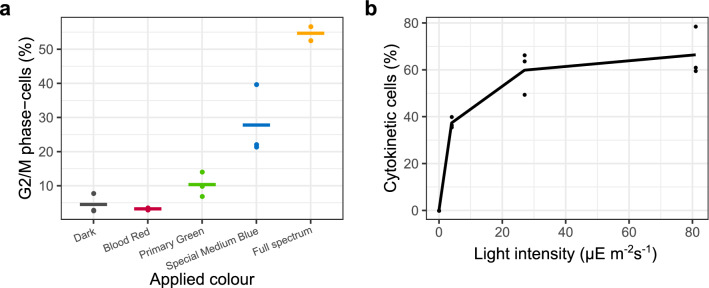
Light requirements for mitotic cell cycle progression (**a)** Percentage of cells in G2-M phase of the cell cycle after 9 h of illumination with different light spectra or darkness, determined with flow cytometry using SYBR green. Points represent individual replicates, while horizontal lines show the average for each condition. (**b)** Proportion of cytokinetic cells at different light intensities, measured 12 h after the end of dark arrest. In the dark, no cytokinetic cells were observed. Points represent individual data points while the line connects the average percentage for each intensity.

Dark-synchronized cultures were subjected to different spectra, darkness and full spectrum for 9 h, a time period after which part of the population has moved into the G2 and M phase of the cell cycle^10^. Flow cytometric measurements of the proportion of G2/M phase cells showed that only 4.5% and 3.2% of the cells passed the S phase in the dark and red light respectively (Fig. 3a). All other treatments showed significantly increased G2/M levels compared to red light (*p* < 0.05), with blue and full spectrum light additionally exceeding the level of green light (Fig. 3a, Supplementary Fig. S5). Thus, cell cycle progression after a dark arrest appears to be blue light regulated, as is the case in *P. tricornutum*^58^*.* Furthermore, we assessed population growth by quantifying cytokinetic cells (doublets) under a range of light intensities. No cytokinetic cells were observed in the dark, and the proportion of cytokinetic cells rose sharply with increasing light intensities (*p* < 0.001) and reached a plateau at 27 µE m^−2^ s^−1^ (Fig. 3b, Supplementary Fig. S5). High growth rates at relatively low irradiances have been observed in several benthic diatom species, illustrating the highly efficient light utilization of these diatoms^25–28^. Alternatively, the rapid levelling off at higher irradiances might be due to the cultures being acclimated to low light, since they were maintained at light intensities of 13 µE m^−2^ s^−1^ before the dark arrest.

**Figure 4:**
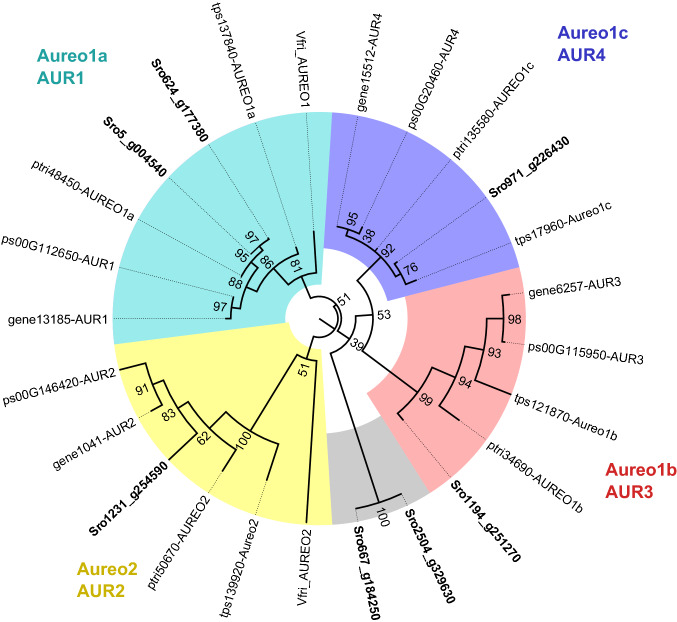
Circular maximum likelihood phylogenetic tree of aureochrome protein sequences. *S. robusta* homologs (“Sro”, in bold) are clustered in relation to previously annotated aureochromes from the diatoms *Phaeodactylum tricornutum* (“ptri”), *Thalassiosira pseudonana* (“tps”), *Fragilariopsis cylindrus* (“gene”) and *Pseudo-nitzschia multiseries* (“ps”) as well as the xanthophyte *Vaucheria frigida* (“Vfri”). The four recognized clades are indicated by colours, and both the clade designation (AUR) and name of the *P. tricornutum* homolog in each clade (Aureo) are indicated at the outside of the tree. Two atypical *S. robusta* aureochromes that did not cluster with one of the clades are indicated in grey. The phylogenetic tree was midpoint rooted and bootstrap values are indicated in each node.

**Figure 5:**
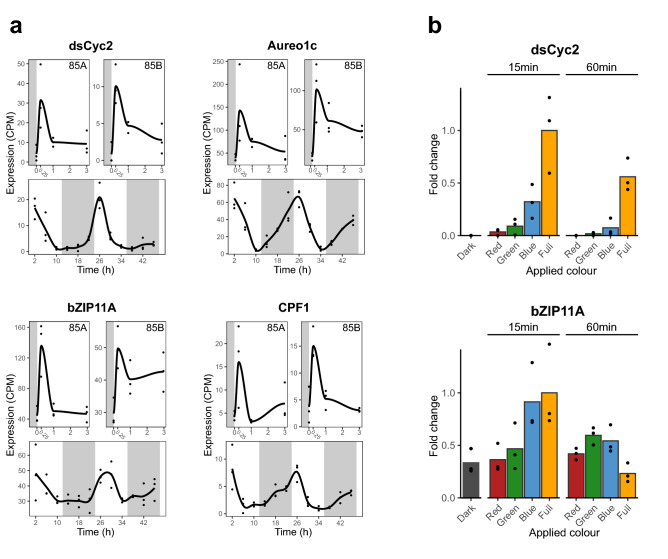
Expression of putative light-dependent G1 checkpoint genes in *Seminavis robusta. *(**a)** Expression over time (in h) in counts per million (CPM) of genes potentially downstream of the blue light photoreceptor *Aureo1a*: diatom-specific cyclin 2 (*dsCyc2*), aureochrome 1c (*Aureo1c*), basic leucine zipper transcription factor *bZIP11A* and cryptochrome/photolyase family 1 (*CPF1*). On top: transcriptome data representing the response to light after a prolonged dark arrest from Moeys et al. (2016) (strain 85B)^7^ and Bilcke et al. (2021a) (strain 85A)^10^. Below: expression throughout a 2-day time series in a 12/12 day/night rhythm from Bilcke et al. (2021b)^62^. Grey shading represents dark conditions. (**b)** RT-qPCR measurements of *dsCyc2* and *bZIP11A* expression in darkness (“Dark”) and after treatment with light spectra for different durations (15 min, 60 min). The Lee colour filters used for these treatments were Primary Green (#139), Blood Red (#789) and Special Medium Blue (#363). Relative expression is given as fold changes compared to the average expression of full spectrum light control after 15 min of illumination. Individual data points are shown as dots, while bar plots represent the average fold change for each treatment.

We identified homologs of the *P. tricornutum* light-dependent checkpoint genes *dsCyc2* and *bZIP10* in the *S. robusta* genome^58^, as well as blue light photoreceptors (aureochromes and *CPF1*) and the transcription factor *bZIP11*, whose transcription was implicated to be regulated by *Aureo1a* blue light perception in *P. tricornutum*^59^ (Supplementary Table S2). The seven aureochrome genes of *S. robusta* were further classified by constructing a maximum-likelihood phylogenetic tree, which revealed two *Aureo1a* homologs, one *Aureo1b, Aureo1c* and *Aureo2* and two atypical aureochromes that did not cluster with any major clade (Fig. 4). Next, we queried the expression of candidate light-dependent checkpoint genes in existing RNA-seq datasets of vegetative cultures from two strains (85A, 85B) being illuminated after a dark arrest^7,10^. Differential expression (DE) analysis of gene expression in the dark versus 15 min of illumination revealed 14,799 significant DE genes in at least one data set (Supplementary Data Set S1). Despite being treated with the same light intensity (80 µE m^−2^ s^−1^), the number of DE genes was about two-fold higher in strain 85A compared to strain 85B. Additionally, rhythmic gene expression of candidate genes over diurnal cycles was retrieved from a 12 h/12 h light/dark diurnal RNA-seq study^62^. As the first time point is situated 2 h after dawn, this data set does not profile the immediate transcriptional responses to light onset but rather shows the transcriptional dynamics in a diel rhythm. The *S. robusta dsCyc2* homolog showed the same characteristic expression profile as in *P. tricornutum*, being significantly upregulated by 15 min of light and peaking at dawn in the diurnal cycle (Fig. 5a, Supplementary Table S3). *Aureo1c*, *bZIP11A* and *CPF1* had a similar response to light onset, suggesting their regulation by *Aureo1a* is conserved as well^59^ (Fig. 5a). Notably, *Aureo1c* and *CPF1* showed anticipatory expression before the first onset of light in the diurnal experiment, pointing towards a circadian element in the regulation of their transcription (Fig. 5a). In contrast to these other elements, the expression of the photoreceptor *Aureo1a* was not maximal just after light onset, as one homolog phased at late night and the other at midday (Supplementary Table S3, Supplementary Fig. S6). The *Aureo1a1* homolog was even significantly downregulated by 15 min of light in both strains (Supplementary Table S3), a response that has also been observed in *P. tricornutum*^59,63^.

To verify blue light photoreceptor dependent expression of *dsCyc2* and *bZIP11A*, we quantified their expression with real-time quantitative PCR (RT-qPCR) over the course of a time series of 0 h (darkness), 15 min and 60 min, during which cultures were exposed to red, blue, green and full spectrum light. Fifteen minutes of white and blue light caused a significantly upregulation of both *dsCyc2* and *bZIP11A* compared to darkness (*p* < 0.05), while red and green light did not cause such an induction (Fig. 5b, Supplementary Fig. S7). The blue-light response of *dsCyc2* was most pronounced, with expression being almost undetectable in darkness and in red light, while white and blue light induced a highly significant upregulation (Fig. 5b).

## Discussion

**Figure 6:**
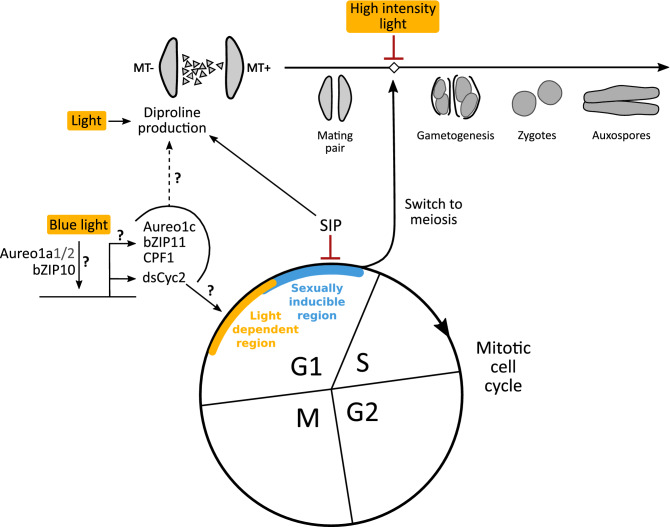
A hypothetical model for the influence of light on the vegetative and sexual life cycle of *Seminavis robusta*. On top, the different sexual stages of the *S. robusta* mating process are shown. Below, the mitotic cell cycle is visualized, with positions of the light-dependent checkpoint (yellow) and sexually inducible region (blue) in the G1 phase. Genes presumably involved in the progression of the G1 light-dependent checkpoint under blue light are indicated, with an aureochrome *Aureo1a* homolog (either *Aureo1a1* or *Aureo1a2*) and bZIP10 driving the expression of the diatom-specific cyclin *dsCyc2* and other putative downstream genes (*Aureo1c*, basic leucine zipper *bZIP11*, cryptochrome/photolyase family *CPF1*). A red flat ended line represents inhibition of a particular step, while black arrows indicate induction or progression. SIP = sex inducing pheromone. MT = mating type. Hypothetical steps that require further elucidation are indicated with question marks. The dotted-line arrow shows possible pathways for light-dependent regulation of diproline production via targets of Aureo1a.

Diatom sexual reproduction has rarely been observed in the natural environment^64–67^ and most recent studies have focused on the physiological and genetic mechanisms underlying sex^6,8–10,68,69^. The relationship between external conditions and sex has received little attention in recent years. Sexual reproduction in homothallic centric diatoms below the SST can often be induced by a change in environmental conditions, such as nutrient concentration, salinity, temperature and light intensity^39,40,48,70–72^. In contrast, sexual reproduction in pennate diatoms depends on the presence of a partner from the compatible mating type^3^. Increasing evidence, however, shows that both biotic and abiotic factors can influence the success of sexual reproduction in pennate diatoms. These include the presence of associated bacteria^73^, inorganic nutrients^74^ and the spectral composition and magnitude of the incident light^28,45^. Here, we showed that the light regime is vital for mating in the model pennate diatom *S. robusta*. Notably, different stages of the mating process had different requirements in terms of light intensity and spectrum (Fig. 6).

The first microscopically discernable stage of the *S. robusta* mating process is the mating pair, which is the result of movement of MT+ towards the source of the attraction pheromone diproline^7^. Vigorous mating pair formation in *S. robusta* occurred across a broad range of light intensities, ranging from 4 to 81 µE m^−2^ s^−1^. A sizeable proportion of pairs was already observed under a low intensity of 4 µE m^−2^ s^−1^, suggesting the action of a photoreceptor. Indeed, further experimentation showed that blue light and to a lesser extent green light supports mating pair formation, whereas red light does not. Next, we showed that these light conditions are similar to those required for progression through the light-dependent checkpoint of the mitotic cycle. Therefore, we hypothesize that the dependence of pair formation on blue light is caused by the activity of the light-dependent checkpoint in the G1 phase. This checkpoint would regulate the progression into the sexually inducible cell cycle phase, where cells commit to mating if a partner is found through pheromone communication^7,10^ (Fig. 6). The downstream position of the sexually inducible region relative to light perception can be deduced from the ability of SIP administered at the end of a dark arrest to still induce a cell cycle arrest^10^. The rapid onset of sexual reproduction after illumination further supports the idea that the sexually inducible region is situated directly downstream of the light dependent region, similar to the situation in the diatoms *T. weissflogii* and *N. lanceolata*^28,44^. All known molecular components of the light-dependent cell cycle pathway of *P. tricornutum*^58,59^ were identified in *S. robusta*. The expression profile of the two *Aureo1a* homologs differed markedly, but the functional relevance of this duplication needs further study*.* The almost instantaneous transcriptional activation of the *S. robusta dsCyc2* homolog following blue light exposure might suggest that the *P. tricornutum* pathway underlying the light-dependent checkpoint is conserved. Thus, the action spectrum of mating pair formation might be explained by an *Aureo1a* blue-light photoreceptor homolog inducing the transcription of *dsCyc2*, which in turn causes progression into the sexually inducible region of the cell cycle (Fig. 6). Despite our efforts, a mechanistic understanding of how cell pairing is inhibited in the absence of blue light is still lacking. A possible explanation is that diproline production by MT- is restricted to the sexually inducible region of the G1 phase. This hypothesis is supported by the lack of diproline production in the dark^6^, and by the quick and transient upregulation of diproline biosynthetic genes in SIP + treated MT- cultures after release from a dark arrest in G1 phase^10^. Alternatively, blue light perception might be upstream of searching behavior in MT+, or may be required for cell–cell recognition to stabilize the nascent cell pair. The situation might be more complicated than suggested by these simple models. For example, different blue light photoreceptors might be linked to responses such as pheromone production and cell cycle progression into the sexually inducible region. It is clear that more work is needed to unravel the exact mechanisms that connect sex pheromone signaling, cell cycle and the light spectrum with reproductive output in this species.

Following successful pairing, each gametangium in a mating pair gives rise to two gametes (gametogenesis), which eventually fuse to form zygotes. In contrast to pair formation, gametogenesis was most successful at low irradiances of 4 to 10 µE m^−2^ s^−1^ (Fig. 6). Higher intensities largely inhibited the transition of paired cells into gametes, resulting in paired cells that are arrested in their development still dominating the crossed culture after 16 h. A possible explanation of the sensitivity to excess light during gametogenesis lies in the filtering of ultraviolet (UV) light by the diatom frustule^75^. Diatoms shed their parental frustule during gametogenesis and spend their gamete and zygote stage largely unprotected^4^, which might make them more vulnerable to detrimental UV levels^75^. However, this scenario is hard to reconcile with reports of high light intensities triggering auxosporulation in centric diatom species^39,42^, as well as planktonic pennates from the genus *Pseudo-nitzschia* producing gametes and auxospores close to the water column surface and in the surf zone^66,67^. Alternatively, restriction of gametogenesis under high light may be an adaptation specific to benthic raphid diatoms because the largely motionless gametes, zygotes and auxospores cannot make use of behavioral photoprotection, making them prone to light stress. Indeed, the benthic raphid diatom *H. ostrearia* exhibits a similar preference for low irradiances during sexual reproduction^45^. In contrast, however, the benthic pennate *N. lanceolata* showed high proportions of sexualized cells at 96 µE m^−2^ s^−1^ ^28^_._

The formation of auxospores, the final stage of the mating process, mirrors the light requirements which were encountered during mating pair formation and gametogenesis, i.e. auxospores were most abundant under a blue light source and low intensity conditions. This suggests that zygotes progress through the remainder of the mating process unhindered, without further restrictive light checkpoints.

The dependency of mating on blue light (and to a lower extent green light) in *S. robusta* and *C. didymus*^50^ is in line with a general propensity of blue light to regulate various cellular processes in diatoms, such as cell cycle progression, phototaxis, vertical migration and production of metabolic compounds such as the pigment marennine^32,33,36,37,58,76^. The identification of functional red-light phytochromes in diatoms^19^ and species-specific red light responses in motility and sinking^37,38,77^ has recently challenged this perspective, suggesting that red light sensing plays a more important role in diatoms than was previously anticipated. Red light sensing is thought to play a role in surface layers or in turbid waters where blue light is absorbed by CDOM or phytoplankton. Alternatively, it may serve as a biotic signal sensor, picking up red-shifted chlorophyll fluorescence from neighboring benthic algae^19,78^. Nonetheless, the preference to red light for auxosporulation in the diatom *H. ostrearia* is puzzling, given that this motile benthic diatom is phylogenetically closely related to *S. robusta*, both belonging to the Naviculaceae^45,79^.

In conclusion, sexual reproduction in *S. robusta* relies on a complex interplay of cell size, sex pheromone signaling, cell cycle progression and the perception of light (Fig. 6). The identification of these light requirements improves our insight into the mating process of *S. robusta*, which has become one of the leading genetic model organisms for the diatom life cycle. Although the dependency on blue light may be conserved in species where induction of sex also depends on a G1 phase light checkpoint such as *T. weissflogii*^44^, the model presented here for *S. robusta* is not readily extendible to other diatom species. Not only the environmental conditions inducing sex but also strategies for mate finding are highly diverged in diatoms, even among raphid pennate diatoms such as *S. robusta* and *P. multistriata*^9^*.* Hence, the optimal light regime and underlying genetic pathways for other life cycle model species will have to be determined on a species-by-species basis. The few reports of planktonic diatoms auxosporulating in natural samples are associated with different seasons (spring, summer or autumn)^64–67,70,80^, suggesting that sexualization is triggered by the perception of external conditions. However, these sightings are almost invariably associated with the end of a bloom, so their induction is more likely triggered by nutrient depletion or quorum sensing than by light. Observations of sexual stages in natural samples are still lacking for *S. robusta* and most other benthic diatom species. Therefore, it is difficult to link experimentally determined cues with the ecological conditions under which sex occurs in nature. The fact that sexualization in *S. robusta* under our laboratory conditions was dependent on low irradiances suggests that sexual reproduction in nature is either linked to vertical migration into low light conditions deeper in the biofilm or sediment, is restricted to autumn or winter, or is induced during periods of turbidity, e.g. during macroalgal or phytoplankton blooms or when high levels of resuspended sediment are present. A promising alternative approach would constitute quantifying diatom sex-specific gene expression in oceanic metatranscriptome datasets such as TARA oceans^81^. The distribution of such marker genes could then be correlated to environmental conditions at each sampling station to gain insight into the conditions required for sex.

## Methods

### Strains and culture conditions

*Seminavis robusta* strains PONTON36 (MT+, DCG 0462) and PONTON34 (MT-, DCG 0460) with a cell size below the SST (< 50 µm) were obtained from the Belgian Coordinated Collection of Microorganisms (BCCM/DCG, http://bccm.belspo.be/about-us/bccm-dcg) and were used for all crossing experiments. For experiments to monitor the mitotic cell cycle, PONTON36 below the SST was used. Cultures were grown in artificial sea water as detailed in Bilcke et al. (2021b)^62^, supplemented with Guillard’s F/2 enrichment solution and made axenic by adding 400 mg L^−1^ ampicillin, 400 mg L^−1^ penicillin, 100 mg L^−1^ streptomycin and 50 mg L^−1^ gentamicin^82^. By default, cultures were grown in a climate-controlled plant growth chamber (Weiss Technik) at a temperature of 21 °C and using a light intensity of 13 µE m^−2^ s^−1^ in a 12 h:12 h day/night cycle, unless stated otherwise. An overview of experiments, strains, conditions and time points used in this paper can be found in Table 1.

**Table 1:** Overview of experiments (rows) included in this study.

N°	Fig.	Response	Cue	Strain(s)	Levels	Time point(s)	n
1	1a	Mating	Light intensity (1)	PONTON36 PONTON34	0, 4, 10, 27, 81 and 108 µE m^−2^ s^−1^	14 h	3
2	1a	Mating	Light intensity (2)	PONTON36 PONTON34	0, 4, 10, 27, 81 and 108 µE m^−2^ s^−1^	14 h	3
3	1b	Mating	Light intensity (time series)	PONTON36 PONTON34	4, 27 and 81 µE m^−2^ s^−1^	10 h, 12 h, 14 h, 16 h	2
4	2	Mating	Spectrum	PONTON36 PONTON34	Red: #789 and #787 Green: #139 Blue: #183 and #363 Control: full spectrum, darkness	14 h	3
5	3a	Mitotic S phase	Spectrum	PONTON36	Red: #789 Green: #139 Blue: #363 Control: full spectrum, darkness	9 h	3
6	3b	Cell division	Light intensity	PONTON36	0, 4, 27 and 81 µE m^−2^ s^−1^	12 h	3
7A	5a	RNA-seq expression Bilcke et al. (2021a)^10^	Light onset after dark arrest	85A	Dark arrest, light onset (control conditions of Bilcke et al. 2021a)	Dark, 15 min, 1 h, 3 h	3
7B	5a	RNA-seq expression Moeys et al. (2016)^7^	Light onset after dark arrest	85B	Dark arrest, light onset (control conditions of Moeys et al. 2016)	Dark, 15 min, 1 h, 3 h	3
7C	5a	RNA-seq expression Bilcke et al. (2021b)^62^	Diurnal cycle	85A	12 h light: 30 µE m^−2^ s^−1^ 12 h darkness	2 h, 6 h, 10 h, 14 h, 18 h, 22 h, 26 h, 30 h, 34 h, 38 h, 42 h, 46 h	3
8	5b	RT-qPCR expression of *dsCyc2* and *bZIP11*	Spectrum	PONTON36	Red: #789 Green: #139 Blue: #363 Control: full spectrum, darkness	Dark, 15 min, 60 min	3

The column “Fig.” indicates the figure where results of each experiment are presented. “Response” gives the response that was quantified, while “Cue” and “Levels” contain the type and treatment levels of the experimental condition (for spectral experiments, the Lee Filters IDs are given). “Strain(s)” shows the *S. robusta* strain that was used for each experiment. “Time point” indicates the duration of light treatment after which response was quantified. The number of replicates per treatment/time point is shown in column “n”. Data for expression analyses (N° 7) was retrieved from existing transcriptomic studies, as shown in the “Response” column.

### Standardized crossing and scoring of sexual stages

Cultures of each mating type were grown in large Cellstar culture flasks (175 cm^2^, Greiner Bio-One) at a surface density between 4000 and 5400 cells per cm^2^. After 48 h of growth to allow for accumulation of SIP in the medium, cultures were subjected to an extended dark period of 24 h to synchronize their cell cycle in G1 phase^56^. Just before the end of the dark treatment, cultures were suspended in their medium by scraping and 10 mL of each mating type was pooled in a small Cellstar culture flask (25 cm^2^, Greiner Bio-One), which was placed in continuous light. The proportion of sexual stages in each flask was determined by taking multiple random microscopic pictures of each flask using a Primovert inverted microscope at 20 × magnification, followed by scoring with the cell counter plugin in FIJI (ImageJ). Mating stages were typically scored at a reference time point of 14 h post-illumination, which showed high proportions of sexual cells in pilot experiments. For the intensity time series experiment, mating stages were determined after 10 h, 12 h, 14 h and 16 h of illumination. The following sexual cell stages were identified: paired cells (individual cells in a mating pair), gametes & zygotes, auxospores, and large cells (initial cells or progeny)^83^. Gametes and zygotes were classified as a single category as they are not easily distinguishable. To model count data from crossing experiments representing one discrete time point, we adopted generalized linear models (GLM) with a quasibinomial distribution to account for overdispersion. When one of the conditions contained 0 counts in all replicates, an offset count of 1 was added to all samples to improve fitting. Post-hoc pairwise comparisons were carried out using the Wald-tests implemented in the glht function of the multcomp package, testing for all possible pairwise comparisons with mcp(treatment = “Tukey”)^84^. P-values were adjusted for multiple testing with the single-step method, adopting a family-wise error rate of < 0.05 for statistical significance^84^. Time series counts were modelled using quasibinomial logistic regression curves and differences in their intercept at 10 h and slope coefficients were evaluated with t-tests compared to the low intensity (4 µE m^−2^ s^−1^) reference treatment level, using the glm() procedure in R^85^. Results of all statistical tests can be found in Supplementary Data Set S2.

### Assessing vegetative cell division

The effect of different light intensities on population growth was determined by quantifying the occurrence of cytokinetic cells after 12 h of illumination, a time point at which high proportions of cytokinetic cells were observed in preliminary experiments. In each small flask, multiple random microscopic pictures were taken with a Primovert inverted microscope at 20 × magnification with the cell counter plugin in FIJI (ImageJ). Cytokinetic cells (also called doublets) were scored as cells showing a visible, high-contrast cleavage furrow separating the still connected daughter cells, as defined by Gillard et al*.* (2008)^56^. The proportion of dividing cells was modelled using GLMs with a quasibinomial distribution, and Tukey’s pairwise comparisons were performed using Wald tests with the multcomp package^84^.

### Quantification of S phase progression

In order to verify the existence of a blue-light checkpoint in the G1 phase of the cell cycle, progression through the premitotic S phase under different spectra was assessed with flow cytometry. Cells were harvested by scraping after 9 h of illumination. Then, suspended cells were pelleted by centrifugation (5 min at 3000 rpm) and the supernatant was discarded. Cells were subsequently fixed in 10 mL ice cold 75% ethanol and later resuspended in 1 mL of ice-cold 75% ethanol and rinsed three times with 1 mL of phosphate-buffered saline buffer (PBS). After 20 min of RNAse treatment at 37 °C, the sample was centrifuged and the pellet was resuspended in 1 mL of 1 × SYBR green solution in PBS. After 10 min of incubation, SYBR green fluorescence was measured on a Bio-Rad S3e cell sorter. Flow cytometry analysis was performed using the flowCore and ggcyto packages for R. The cell population was gated based on their forward scatter and side scatter profile and G1 vs G2/M phase cells were gated based on the positions of maxima in the FL1 histogram. The proportion of G2/M phase cells relative to the total set of cells (G1 + G2/M) was modelled using generalized linear models with a quasibinomial distribution, and Tukey’s pairwise comparisons were performed through Wald tests with the multcomp package^84^.

### Properties of the light source

Illumination for all experiments was provided by fluorescent lamps (Spectralux Plus NL-T8, Radium). The reference spectral composition and downwelling plane irradiance of the experimental setup were measured with a PAR200 Quantum Spectrometer (UPRtek). During the experiments, the incident light intensity at each setup (distance, optical filter) was measured with a Testo 435 Illuminance meter equiped with lux probe N° 0635 0545. In this work, light intensity is reported in moles of photons (Einstein, E) per unit area (m^2^) and time (s), i.e. the photon flux density (PFD). To convert from illuminance to PFD, the spectral properties of the lamp and optical filters were used to calculate conversion factors (Eq. 1).

1 $$K_{n} ~ = ~\frac{{PFD}}{I}~ = \frac{{10^{{ - 3}} }}{{N_{a} hc}}~\frac{{\mathop \smallint \nolimits_{{380}}^{{780}} T_{n} \left( \lambda \right)~E_{d} \left( \lambda \right)~\lambda ~d\lambda }}{{K_{m} \mathop \int \nolimits_{{380}}^{{780}} T_{n} \left( \lambda \right)~\bar{y}\left( \lambda \right)~E_{d} \left( \lambda \right)~d\lambda }},$$

where *I* is the illuminance (lm m^−2^), *N*_a_ is the Avogadro constant (mol^−1^), *h* is the Plank constant (J s), *c* is the speed of light in vacuum (m s^−1^), *K*_m_ is the maximum luminous efficacy constant (683 lm W^−1^), *T*_*n*_ is the transmittance relative to air of the setup *n*, *y* is the luminosity function of the average photopic observer^86^, *E*_d_ is the spectral plane irradiance (W m^−2^ nm^−1^) and $\lambda$ is the wavelength (nm). The conversion factors *K*_n_ have units of µE lm^−1^ s^−1^. Treatments with full spectrum have a spectral constant *T*_n_ = 1. The spectral properties of the lamp, filters and conversion factors are provided in the supplementary material (Supplementary Fig. S2 and S3, Supplementary Table S1).

### Light intensity experiments

To assess the effect of light intensity on sexual reproduction, cultures were subjected to a range of PFD’s, ranging from dark to a moderate-high intensity of 108 µE m^−2^ s^−1^ (Table 1). The incident PFD was varied by placing cultures at different distances to the light source and covering flasks with translucent white paper. Dark control cultures were covered with aluminum foil. All experiments were carried out in triplicate, except for the time series experiment which has two replicates per intensity and time point.

### Light spectrum experiments

Before transfer to light with an incident intensity of 61 µE m^−2^ s^−1^, dark-arrested cultures in small flasks were covered with colour filters (Lee Filters, UK): Blood Red (#789), Marius Red (#787), Primary Green (#139), Moonlight Blue (#183) and Special Medium Blue (#363). For the flow cytometry and qPCR experiments, a reduced set of filters was used, consisting of Blood Red, Primary Green and Special Medium Blue (Table 1). The transmittance spectrum of each filter (Supplementary Fig. S2) was measured using a Lambda-650S spectrophotometer equipped with an integrating sphere (PerkinElmer). To reduce the potential confounding effect of light intensity on the spectral composition experiments, an approximate correction was made to compensate for the transmittance in the spectral range of each colour filter. The objective was to provide approximately the same incident light intensity, in the wavelength range of the colour filter, for the colour filter treatment versus the control receiving the full spectrum (Supplementary Fig. S3). The approximate correction was made by changing the illumination to compensate for the transmittance at the wavelength of maximum transmittance of each colour filter. Dark and light controls were included by covering flasks with aluminum foil or leaving them uncovered. All experiments were carried out in triplicate.

### Phylogenetic analysis of aureochromes

Seven *S. robusta* genes were found that belong to the aureochrome family HOM02SEM000300 in the PLAZA Diatoms 1.0 genomic database^20^. To characterize the different classes of aureochrome photoreceptors in *S. robusta*, a phylogenetic analysis was performed, combining *S. robusta* aureochrome protein sequences with a set of aureochrome sequences that were previously annotated in the diatoms *P. tricornutum*, *Fragilariopsis cylindrus*, *T. pseudonana* and *Pseudo-nitzschia multiseries*^11,60^, as well as the aureochromes that were originally discovered and annotated from the xanthophyte *Vaucheria frigida*^18^. *V. frigida* aureochrome1 and aureochrome2 protein sequences were retrieved from NCBI, all other sequences from PLAZA Diatoms. A multiple sequence alignment was performed with MAFFT v7.453 after which poorly aligned positions (-gt 0.75) were trimmed using Trimal v1.4.1^87,88^. A maximal likelihood phylogenetic tree with 1000 ultrafast bootstrap replicates was constructed using IQ-TREE v1.7.0^89^, with the command -mset JTT,LG,WAG,Blosum62,VT,Dayhoff -mfreq F -mrate R.

### Transcriptomic profiling of the expression of candidate light-dependent checkpoint genes

To investigate the transcription of candidate light-dependent G1 phase checkpoint genes, existing *S. robusta* RNA-seq datasets which cover the synchronized dark-to-light transition after a dark arrest were re-analyzed (Table 1). Paired-end Illumina reads from the control condition (no pheromone treatment) were retrieved from Moeys et al. (2016)^7^ and Bilcke et al. (2021a)^10^, consisting of four time points in triplicate: 0 h (dark), 15 min, 1 h and 3 h. Additionally, raw transcriptomic reads from a 48 h diurnal 12 h/12 h light/dark study in *S. robusta* were retrieved for visualization of the diel progression of candidate genes^62^ (Table 1). For all samples, reads were mapped with Salmon v0.9.1^90^ to the longest isoform of *S. robusta* gene models including untranslated regions (UTRs), belonging to the *S. robusta* reference genome version 1.2 ^20^. Mapped reads were imported into R using tximport v1.16.1 ^91^. For visualization of expression levels, the normalized metric counts per million (CPM) was calculated with the EdgeR package^92^. In addition to plotting of expression with ggplot2 ^93^, the response of candidate genes to the dark-to-light transition was investigated by performing DE analysis on the first control time point (15 min) versus dark control (t0) of both dark synchronized datasets^7,10^. For DE analysis, lowly expressed genes were removed by retaining genes for which CPM > 1 in at least 3 samples over both data sets (6 + 6 samples). EdgeR was used to perform TMM normalization on raw reads, after which negative binomial generalized linear models were fitted and differential expression was inferred using likelihood ratio tests. To select genes that were significantly differentially expressed in at least one data set, the two p-values were aggregated using the Sidak method, based on the minimum p-value. The Benjamini–Hochberg procedure was performed on these aggregated p-values, and genes were considered differentially expressed on a 5% false discovery rate (FDR) level (Supplementary Data Set S1). The differential expression results were further enriched with the phase (time point with maximal expression) of each gene in the diurnal RNA-seq dataset, retrieved from Bilcke et al. (2021b)^62^. We specifically focussed on *S. robusta* homologs of genes implied in the light-dependent checkpoint of *P. tricornutum*, namely *dsCyc2*, *Aureo1a*, *Aureo1c*, *bZIP10*, *bZIP11* and *CPF1* (Supplementary Table S3)^58,59^. Homologs of these *P. tricornutum* genes were identified in the *S. robusta* reference genome v1.2^20^ using the PLAZA diatoms v1.0 integrative orthology framework^94^, assigning orthology when genes belong to same orthologous gene family (ORTHO), are phylogenetic tree based orthologs (TROG) and Best Blast Hit and In-paralogs (BHIF) of the *P. tricornutum* candidate genes (Supplementary Table S2).

### Expression quantification under different light spectra using RT-qPCR

For RT-qPCR of the genes *dsCyc2* and *bZIP11*, triplicate cultures treated with a dark arrest were subjected to different colour spectra and a full spectrum control (Table 1), and were subsequently harvested after 15 min and 1 h of illumination. A control t0 sample consisted of dark arrested cells. Cultures were harvested by scraping cells from the surface, which were subsequently collected on a Versapor filter which was flash-frozen in liquid nitrogen. Cell lysis and RNA extraction were performed following the protocol described in Bilcke et al. (2021b)^62^. cDNA was synthesized with the iScript cDNA Synthesis Kit (Bio-Rad). Relative expression levels were determined using a SYBR green LightCycler 480 RT-PCR System (Roche). The stably expressed genes based on the *S. robusta* expression atlas^20^ Sro31_g020410 (protein kinase, Norm1), Sro70_g039100 (NSF attachment protein, Norm2) and Sro397_g134440 (sorting Nexin, Norm3) were used for normalization. Relative expression in terms of fold changes compared to the average expression in 15 min of full spectrum light was calculated using the 2^−ΔΔCt^ method^95^. Primer sequences can be found in Supplementary Data Set S3. Linear models were fitted on log transformed fold changes (logFC) after adding a small offset of 0.01 to each fold change to account for fold changes of zero. Then, an Anova omnibus test and pairwise Tukey post-hoc *t*-tests with single-step correction were performed using the multcomp package for R^84^.

## Supplementary Information

Supplementary Information 1.

Supplementary Information 2.

Supplementary Information 3.

Supplementary Information 4.

Supplementary Information 5.

## Data Availability

Differential expression calls, including FDR adjusted p-values and log_2_ fold changes for each data set, are available in Supplementary Data Set S1. The full results of statistical pairwise comparison tests are included in Supplementary Data Set S2 and RT-qPCR primer sequences are included in Supplementary Data Set S3. Raw measurements of spectral properties of the light source and colour filters are included in Supplementary Data Set S4. Homology information of genes mentioned in this work can be retrieved from the PLAZA Diatoms platform for comparative genomics https://bioinformatics.psb.ugent.be/plaza/versions/plaza_diatoms_01/^20^.

## References

[1] Round FE, Crawford RM, Mann DG (1992). The diatoms: Biology & morfology of the genera.

[2] Amato A (2010). Diatom reproductive biology: Living in a crystal cage. Int. J. Plant Reprod. Biol..

[3] Chepurnov VA, Mann DG, Sabbe K, Vyverman W (2004). Experimental studies on sexual reproduction in diatoms. Int. Rev. Cytol..

[4] Kaczmarska I (2013). Proposals for a terminology for diatom sexual reproduction, auxospores and resting stages. Diatom Res..

[5] Poulíčková A, Mann DG, Mann DG (2019). Diatom sexual reproduction and life cycles.

[6] Gillard J (2013). Metabolomics enables the structure elucidation of a diatom sex pheromone. Angew. Chemie - Int. Ed..

[7] Moeys S (2016). A sex-inducing pheromone triggers cell cycle arrest and mate attraction in the diatom *Seminavis robusta*. Sci. Rep..

[8] Sato S, Beakes G, Idei M, Nagumo T, Mann DG (2011). Novel sex cells and evidence for sex pheromones in diatoms. PLoS ONE.

[9] Basu S (2017). Finding a partner in the ocean: molecular and evolutionary bases of the response to sexual cues in a planktonic diatom. New Phytol..

[10] Bilcke G (2021). Mating type specific transcriptomic response to sex inducing pheromone in the pennate diatom *Seminavis robusta*. ISME J..

[11] Depauw FA, Rogato A, D’Alcalá MR, Falciatore A (2012). Exploring the molecular basis of responses to light in marine diatoms. J. Exp. Bot..

[12] Kirk, J. T. O. *Light and photosynthesis in aquatic ecosystems, third edition*. (2010).

[13] Macintyre HL, Kana TM, Geider RJ (2000). The effect of water motion on short-term rates of photosynthesis by marine phytoplankton. Trends Plant Sci..

[14] Holtrop T (2021). Vibrational modes of water predict spectral niches for photosynthesis in lakes and oceans. Nat. Ecol. Evol..

[15] Clementson LA, Wojtasiewicz B (2019). Dataset on the in vivo absorption characteristics and pigment composition of various phytoplankton species. Data Br..

[16] König S, Juhas M, Jäger S, Kottke T, Büchel C (2017). The cryptochrome-photolyase protein family in diatoms. J. Plant Physiol..

[17] Kroth PG, Wilhelm C, Kottke T (2017). An update on aureochromes: Phylogeny – mechanism – function. J. Plant Physiol..

[18] Takahashi F (2007). AUREOCHROME, a photoreceptor required for photomorphogenesis in stramenopiles. Proc. Natl. Acad. Sci. USA.

[19] Fortunato AE (2016). Diatom phytochromes reveal the existence of far-red-light-based sensing in the ocean. Plant Cell.

[20] Osuna-Cruz CM (2020). The *Seminavis robusta* genome provides insights into the evolutionary adaptations of benthic diatoms. Nat. Commun..

[21] Pushkarev A (2018). A distinct abundant group of microbial rhodopsins discovered using functional metagenomics. Nature.

[22] Underwood GJC, Kromkamp J (1999). Primary production by phytoplankton and microphytobenthos in estuaries. Adv. Ecol. Res..

[23] Hope JA, Paterson DM, Thrush SF (2020). The role of microphytobenthos in soft-sediment ecological networks and their contribution to the delivery of multiple ecosystem services. J. Ecol..

[24] Cartaxana P, Ribeiro L, Goessling JW, Cruz S, Kühl M (2016). Light and O_2_ microenvironments in two contrasting diatom-dominated coastal sediments. Mar. Ecol. Prog. Ser..

[25] Cahoon LB, Beretich GR, Thomas CJ, McDonald AM (1993). Benthic microalgal production at Stellwagen Bank, Massachusetts Bay, USA. Mar. Ecol. Prog. Ser..

[26] Palmisano AC (1985). Shade adapted benthic diatoms beneath Antarctic sea ice. J. Phycol..

[27] De La Peña MR (2007). Cell growth and nutritive value of the tropical benthic diatom, Amphora sp., at varying levels of nutrients and light intensity, and different culture locations. J. Appl. Phycol..

[28] Davidovich NA (1998). Transition to sexual reproduction and control of initial cell size in *Nitzschia lanceolata*. Diatom Res..

[29] Tolhurst TJ, Chapman MG, Murphy RJ (2020). The effect of shading and nutrient addition on the microphytobenthos, macrofauna, and biogeochemical properties of intertidal flat sediments. Front. Mar. Sci..

[30] Poulsen NC, Spector I, Spurck TP, Schultz TF, Wetherbee R (1999). Diatom gliding is the result of an actin-myosin motility system. Cell Motil. Cytoskeleton.

[31] Blommaert L, Lavaud J, Vyverman W, Sabbe K (2018). Behavioural versus physiological photoprotection in epipelic and epipsammic benthic diatoms. Eur. J. Phycol..

[32] Barnett A, Méléder V, Dupuy C, Lavaud J (2020). The vertical migratory rhythm of intertidal microphytobenthos in sediment depends on the light photoperiod, intensity, and spectrum: evidence for a positive effect of blue wavelengths. Front. Mar. Sci..

[33] Prins A, Deleris P, Hubas C, Jesus B (2020). Effect of light intensity and light quality on diatom behavioral and physiological photoprotection. Front. Mar. Sci..

[34] Longphuirt SN (2006). Discovery of microphytobenthos migration in the subtidal zone. Mar. Ecol. Prog. Ser..

[35] Saburova MA, Polikarpov IG (2003). Diatom activity within soft sediments: Behavioural and physiological processes. Mar. Ecol. Prog. Ser..

[36] Cohn SA (2016). Analysis of light quality and assemblage composition on diatom motility and accumulation rate. Diatom Res..

[37] McLachlan DH, Brownlee C, Taylor AR, Geider RJ, Underwood GJC (2009). Light-induced motile responses of the estuarine benthic diatoms *Navicula perminuta* and *Cylindrotheca closterium* (Bacillariophyceae). J. Phycol..

[38] Cohn SA (2015). Comparative analysis of light-stimulated motility responses in three diatom species. Diatom Res..

[39] Drebes G (1966). On the life history of the marine plankton diatom *Stephanopyxis palmeriana*. Helgoländer Meeresun..

[40] Werner DD (1971). Entwicklungscyclus mit Sexualphase bei der marinen Diatomee *Coscinodiscus asteromphalus* – III. Differenzierung und Spermatogenese. Arch. Mikrobiol..

[41] Holmes RW (1966). Short-term temperature and light conditions associated with auxospore formation in the marine centric diatom *Coscinodiscus concinnus* W Smith. Nature.

[42] von Stosch HA, Drebes G (1964). Entwicklungsgeschichtliche Untersuchungen an zentrischen Diatomeen IV - Die Planktondiatomee *Stephanopyxis turris* - ihre Behandlung und Entwicklungsgeschichte. Helgoländer Meeresun..

[43] Drebes G. (1977). Sexuality. The Biology of Diatoms.

[44] Armbrust EV, Chisholm SW, Olson RJ (1990). Role of light and the cell cycle on the induction of spermatogenesis in a centric diatom. J. Phycol..

[45] Mouget JL, Gastineau R, Davidovich O, Gaudin P, Davidovich NA (2009). Light is a key factor in triggering sexual reproduction in the pennate diatom *Haslea ostrearia*. FEMS Microbiol. Ecol..

[46] Furnas MJ (1985). Diel synchronization of sperm formation in the diatom *Chaetoceros curvisetum* Cleve. J. Phycol..

[47] Schultz ME, Trainor FR (1968). Production of male gametes and auxospores in the centric diatoms *Cyclotella meneghiniana* and *C. cryptica*. J. Phycol..

[48] Steele RL (1965). Induction of sexuality in two centric diatoms. Bioscience.

[49] Hiltz M, Bates SS, Kaczmarska I (2000). Effect of light: Dark cycles and cell apical length on the sexual reproduction of the pennate diatom *Pseudo-nitzschia multiseries* (Bacillariophyceae) in culture. Phycologia.

[50] Baatz I (1941). Die Bedeutung der Lichtqualität für Wachstum und Stoffproduktion planktontischer Meeresdiatomeen. Planta.

[51] Pawlowski WP, Sheehan MJ, Ronceret A (2007). In the beginning: The initiation of meiosis. BioEssays.

[52] Davidovich NA, Bates SS (1998). Sexual reproduction in the pennate diatoms *Pseudo-nitzschia multiseries* and *P. pseudodelicatissima* (Bacillariophyceae). J. Phycol..

[53] Brzezinski M, Olson R, Chisholm S (1990). Silicon availability and cell-cycle progression in marine diatoms. Mar. Ecol. Prog. Ser..

[54] Vaulot D, Olson RJ, Chisholm SW (1986). Light and dark control of the cell cycle in two marine phytoplankton species. Exp. Cell Res..

[55] Olson RJ, Vaulot D, Chisholm SW (1986). Effects of environmental stresses on the cell cycle of two marine phytoplankton species. Plant Physiol..

[56] Gillard J (2008). Physiological and transcriptomic evidence for a close coupling between chloroplast ontogeny and cell cycle progression in the pennate diatom Seminavis robusta. Plant Physiol..

[57] Huysman MJJ (2010). Genome-wide analysis of the diatom cell cycle unveils a novel type of cyclins involved in environmental signaling. Genome Biol..

[58] Huysman MJJ (2013). AUREOCHROME1a-mediated induction of the diatom-specific cyclin dsCYC2 controls the onset of cell division in diatoms (*Phaeodactylum tricornutum*). Plant Cell.

[59] Mann, M. *et al.* The aureochrome photoreceptor PtAUREO1a is a highly effective blue light switch in diatoms. *iScience* **23**, 101730 (2020). 10.1016/j.isci.2020.101730 PMC7670200 33235981

[60] Schellenberger Costa B (2013). Aureochrome 1a is involved in the photoacclimation of the diatom *Phaeodactylum tricornutum*. PLoS ONE.

[61] Chepurnov VA (2008). In search of new tractable diatoms for experimental biology. BioEssays.

[62] Bilcke, G. *et al.* Diurnal transcript profiling of the diatom *Seminavis robusta* reveals adaptations to a benthic lifestyle. *Plant J.* tpj.15291 (2021). 10.1111/tpj.15291 33901335

[63] Smith SR (2016). Transcriptional orchestration of the global cellular response of a model pennate diatom to diel light cycling under iron limitation. PLoS Genet..

[64] Assmy P, Henjes J, Smetacek V, Montresor M (2006). Auxospore formation by the silica-sinking, oceanic diatom *Fragilariopsis kerguelensis* (Bacillariophyceae). J. Phycol..

[65] D’Alelio D (2010). The time for sex: A biennial life cycle in a marine planktonic diatom. Limnol. Oceanogr..

[66] Holtermann KE, Bates SS, Trainer VL, Odell A, Armbrust EV (2010). Mass sexual reproduction in the toxigenic diatoms *Pseudo-nitzschia australis* and *P. pungens* (Bacillariophyceae) on the Washington coast, USA. J. Phycol..

[67] Sarno D, Zingone A, Montresor M (2010). A massive and simultaneous sex event of two *Pseudo-nitzschia* species. Deep. Res. Part II Top. Stud. Oceanogr..

[68] Russo MT (2018). MRP3 is a sex determining gene in the diatom *Pseudo-nitzschia multistriata*. Nat. Commun..

[69] Ferrante MI (2019). Exploring molecular signs of sex in the marine diatom *Skeletonema marinoi*. Genes (Basel)..

[70] Waite A, Harrison P (1992). Role of sinking and ascent during sexual reproduction in the marine diatom *Ditylum brightwellii*. Mar. Ecol. Prog. Ser..

[71] Godhe A, Kremp A, Montresor M (2014). Genetic and microscopic evidence for sexual reproduction in the centric diatom *Skeletonema marinoi*. Protist.

[72] Schultz ME, Trainor FR (1970). Production of male gametes and auxospores in a polymorphic clone of the centric diatom *Cyclotella*. Can. J. Bot..

[73] Cirri E, Vyverman W, Pohnert G (2018). Biofilm interactions-bacteria modulate sexual reproduction success of the diatom *Seminavis robusta*. FEMS Microbiol. Ecol..

[74] Bondoc KGV (2019). Decision-making of the benthic diatom *Seminavis robusta* searching for inorganic nutrients and pheromones. ISME J..

[75] Ellegaard M (2016). The fascinating diatom frustule - can it play a role for attenuation of UV radiation?. J. Appl. Phycol..

[76] Mouget JL, Rosa P, Vachoux C, Tremblin G (2005). Enhancement of marennine production by blue light in the diatom *Haslea ostrearia*. J. Appl. Phycol..

[77] Fisher AE, Berges JA, Harrison PJ (1996). Does light quality affect the sinking rates of marine diatoms. J. Phycol..

[78] Ragni M, D’Alcalà MR (2004). Light as an information carrier underwater. J. Plankton Res..

[79] Sabir JSM (2018). Phylogenetic analysis and a review of the history of the accidental phytoplankter, *Phaeodactylum tricornutum* Bohlin (Bacillariophyta). PLoS ONE.

[80] Crawford RM (1995). The role of sex in the sedimentation of a marine diatom bloom. Limnol. Oceanogr..

[81] Carradec Q (2018). A global ocean atlas of eukaryotic genes. Nat. Commun..

[82] Koedooder C (2019). Diatom-bacteria interactions modulate the composition and productivity of benthic diatom biofilms. Front. Microbiol..

[83] Chepurnov VA, Mann DG, Vyverman W, Sabbe K, Danielidis DB (2002). Sexual reproduction, mating system, and protoplast dynamics of *Seminavis* (Bacillariophyceae). J. Phycol..

[84] Hothorn T, Bretz F, Westfall P (2008). Simultaneous inference in general parametric models. Biometrical J..

[85] R Development Core Team. A language and environment for statistical computing. *R Foundation for Statistical Computing* https://www.R-project.org (2018).

[86] Mobley, C. D. *Light and Water: Radiative transfer in natural waters (vol. 592)*. (Academic Press, 2004).

[87] Katoh K, Standley DM (2013). MAFFT multiple sequence alignment software version 7: Improvements in performance and usability. Mol. Biol. Evol..

[88] Capella-Gutiérrez S, Silla-Martínez JM, Gabaldón T (2009). trimAl: A tool for automated alignment trimming in large-scale phylogenetic analyses. Bioinformatics.

[89] Nguyen LT, Schmidt HA, Von Haeseler A, Minh BQ (2015). IQ-TREE: A fast and effective stochastic algorithm for estimating maximum-likelihood phylogenies. Mol. Biol. Evol..

[90] Patro R, Duggal G, Love MI, Irizarry RA, Kingsford C (2017). Salmon provides fast and bias-aware quantification of transcript expression. Nat. Methods.

[91] Soneson C, Love MI, Robinson MD (2015). Differential analyses for RNA-seq: transcript-level estimates improve gene-level inferences. F1000Research.

[92] Robinson MD, McCarthy DJ, Smyth GK (2009). EdgeR: A Bioconductor package for differential expression analysis of digital gene expression data. Bioinformatics.

[93] Wickham, H., Navarro, D. & Lin Pedersen, T. *ggplot2: elegant graphics for data analysis* (2009).

[94] van Bel M (2012). Dissecting plant genomes with the PLAZA comparative genomics platform. Plant Physiol..

[95] Livak KJ, Schmittgen TD (2001). Analysis of relative gene expression data using real-time quantitative PCR and the 2-ΔΔCT method. Methods.

