# Supplementary material for: Light intensity and spectral composition drive reproductive success in the marine benthic diatom Seminavis robusta
Source: Sci Rep. 2021 Sep 2;11:17560. doi: 10.1038/s41598-021-92838-0 (PMC8413402; doi:10.1038/s41598-021-92838-0)
Supplement: Supplementary file 1 — Supplementary Information 1. [file 41598_2021_92838_MOESM1_ESM.pdf]

Light intensity and spectral composition drive reproductive success in the marine benthic diatom *Seminavis robusta*

Gust Bilcke, Lore Van Craenenbroeck, Alexandre Castagna, Cristina Maria Osuna-Cruz, Klaas Vandepoele, Koen Sabbe, Lieven De Veylder, Wim Vyverman

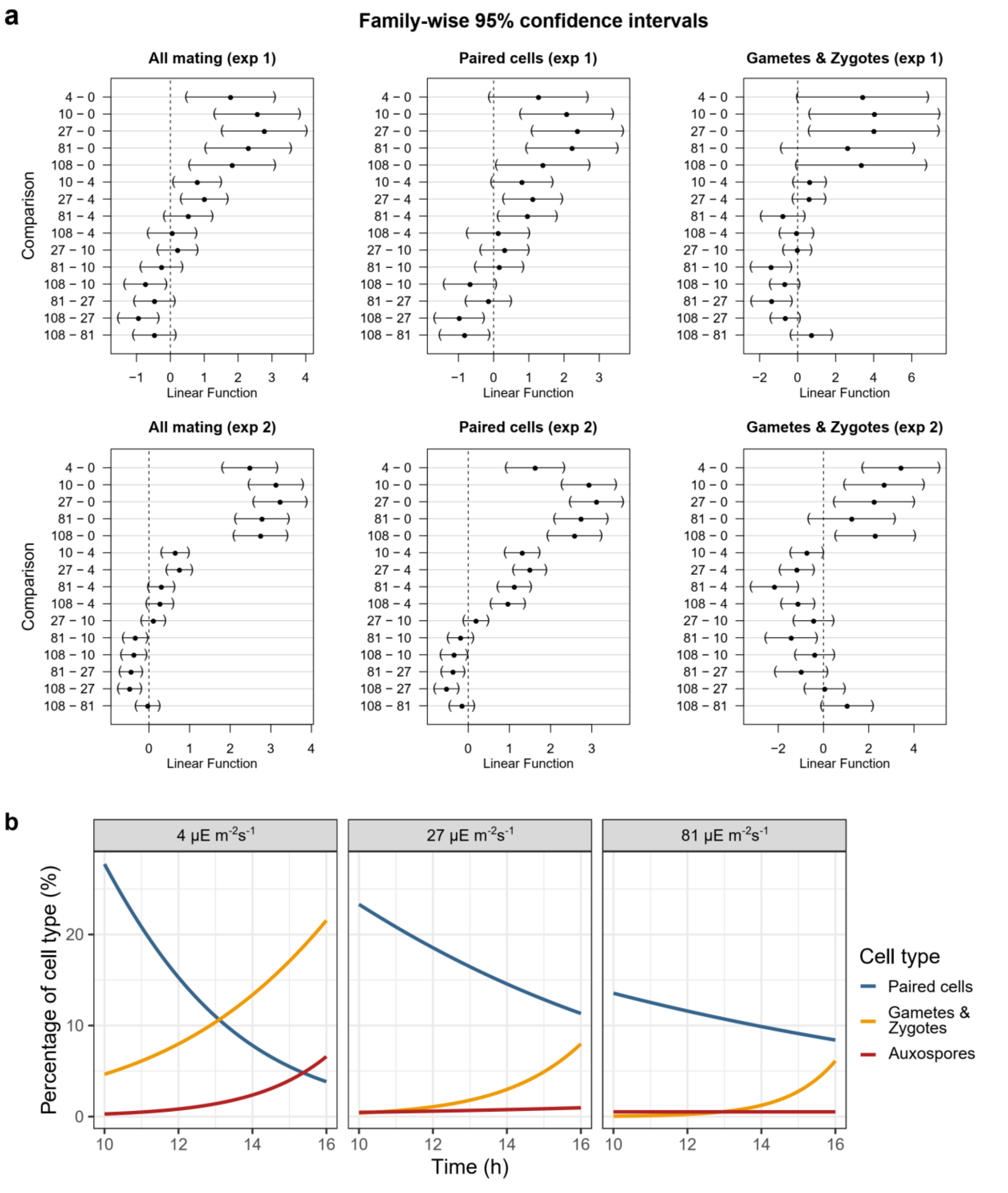

Figure S1: Statistical testing of light intensity mating experiments. **a**: adjusted 95% confidence intervals for Tukey's pairwise comparisons of treatment levels (y-axis numbers represent intensity in µE $m^{-2}s^{-1}$, see Figure 1a). Separate tests were performed for each of the two experimental repeats (exp1, exp2), modelling the sum of all sexual cell stages ("All mating") and separately "Paired cells" and "Gametes & Zygotes". **b:** Sexual cell stage percentages in mixed PONTON36xPONTON34 cultures in function of time since illumination, after treatment with three different light intensities (grey boxes). Lines show the quasibinomial regression curves fitted to the data shown in Figure 1b, with colours representing the cell type.

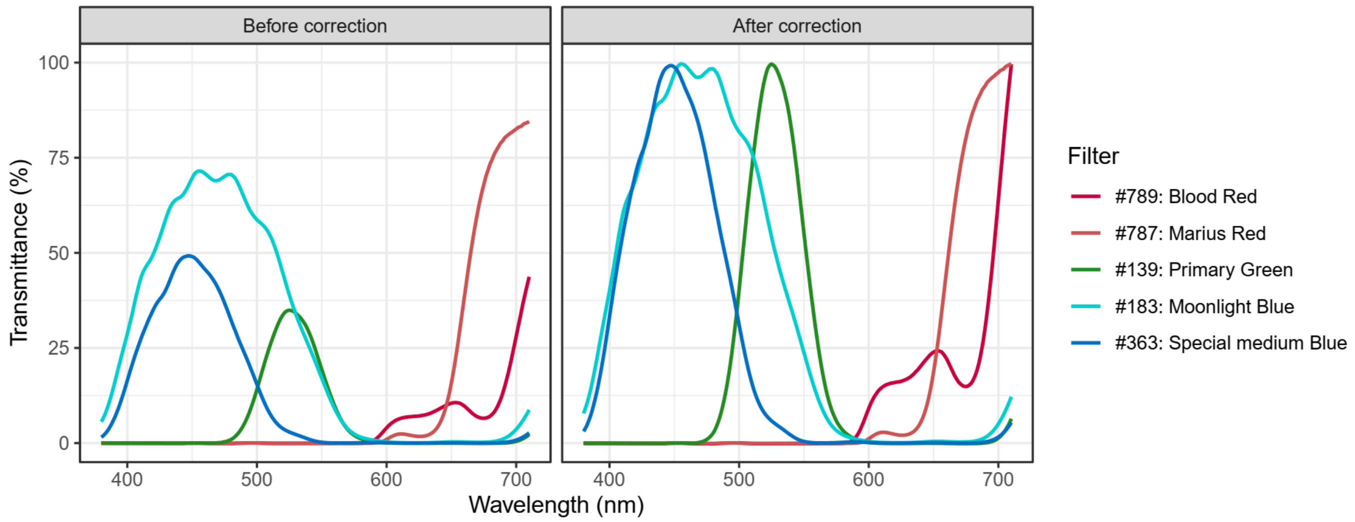

Figure S2: transmission spectrum of colour filters used for light spectrum experiments. The transmittance in percent over the range of photosynthetically active wavelengths (in nm) is shown for different filters used in this study (left). To the right, the transmittance is shown multiplied by the light intensity correction factor that was applied to normalize the transmittance for the wavelength of interest (**Table S1**).

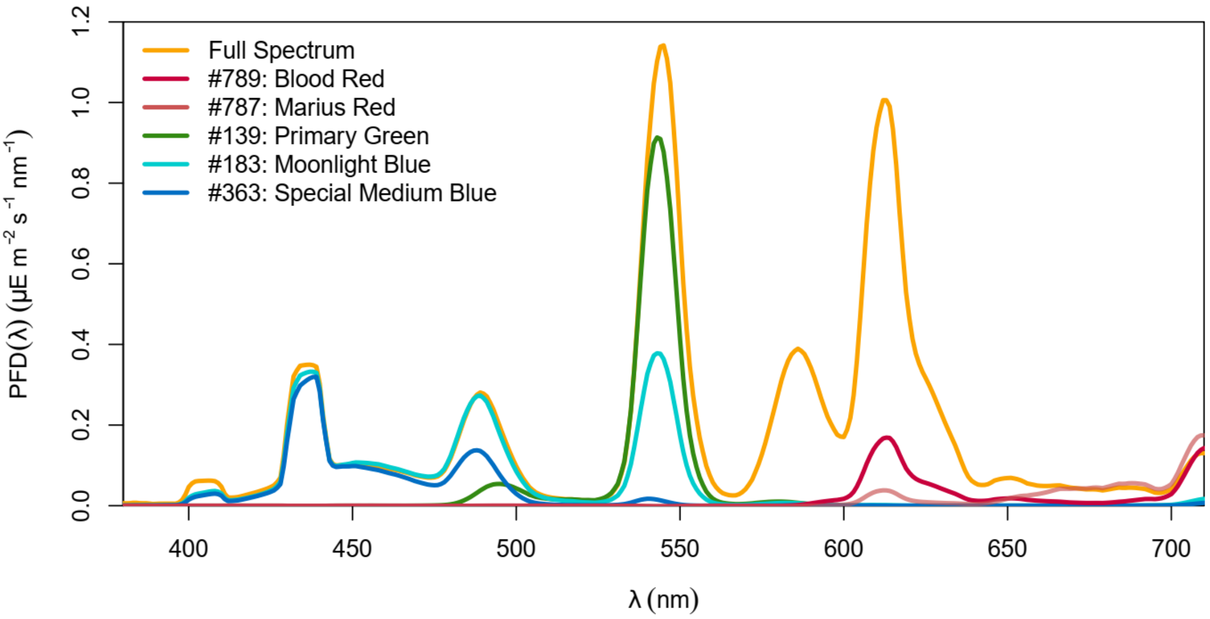

Figure S3: spectral photon flux density for the full spectrum and colored filter experiments. The integral with respect to wavelength in the range 380 nm to 780 nm gives the total received PFD in each experiment (**Table S1**).

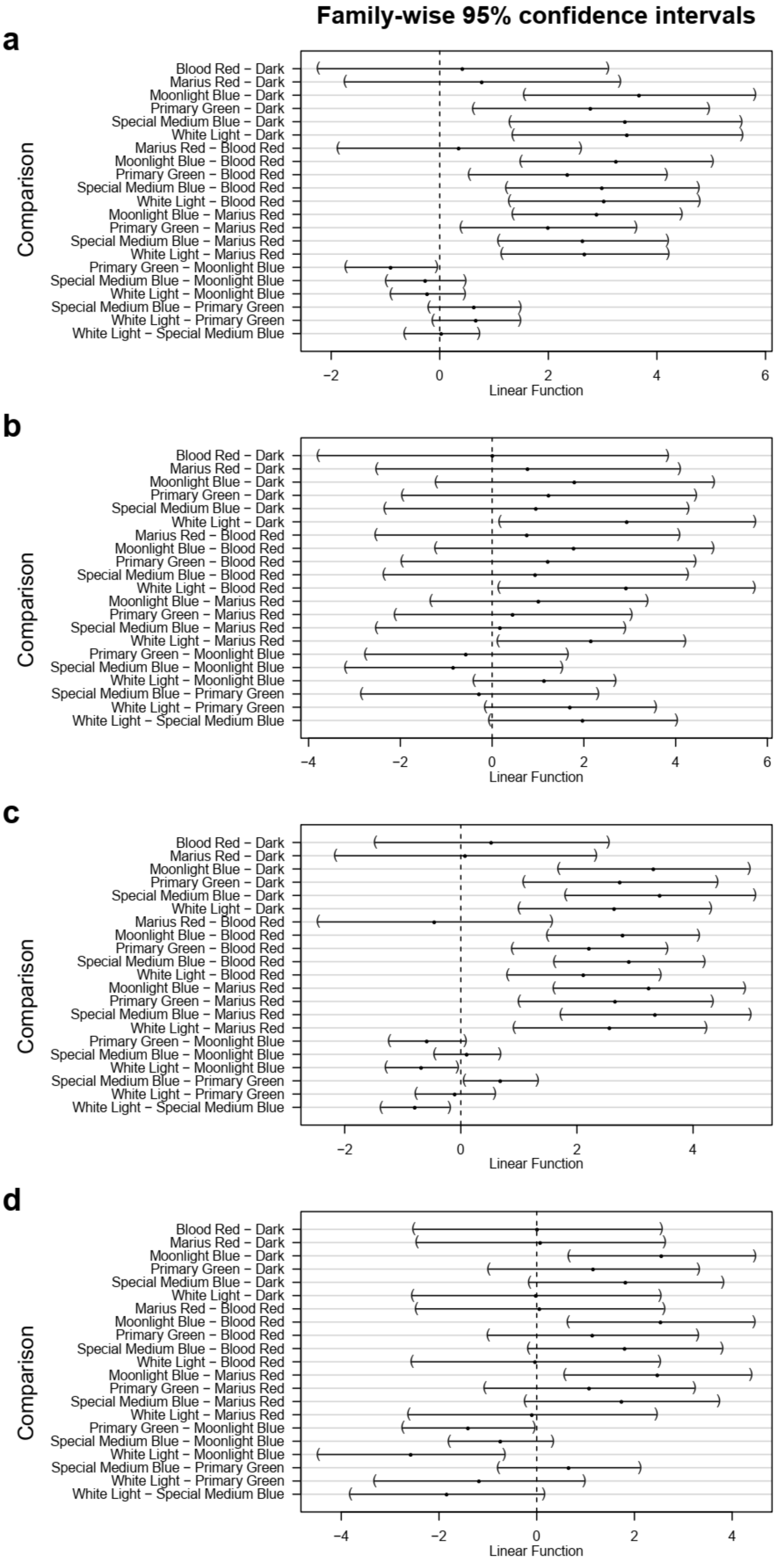

Figure S4: adjusted 95% confidence intervals for Tukey's pairwise comparisons, testing for differences in the prevalence of sexual stages between light spectra treatments (**Figure 2**). **a:** all sexual stages, **b:** paired cells, **c:** gametes & zygotes, and **d:** auxospores.

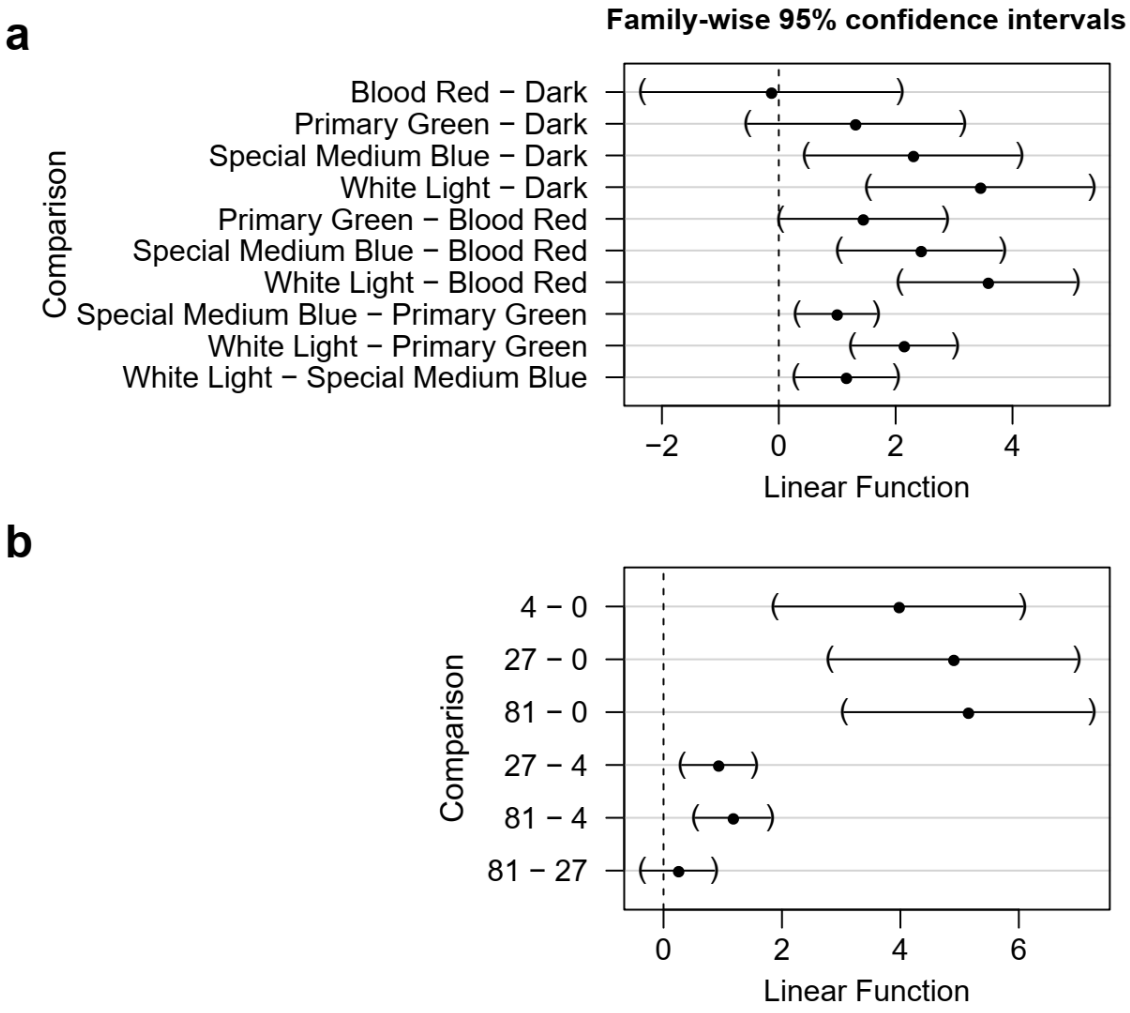

Figure S5: adjusted 95% confidence intervals for Tukey's pairwise comparisons. a: pairwise comparisons of the percentage of G2/M phase cells after treatment with different light spectra for 9h (Figure 3a). **b:** pairwise comparisons of the percentage of cytokinetic cells after treatment with different light intensities (in µE $m^{-2}s^{-1}$) for 12h (Figure 3b).

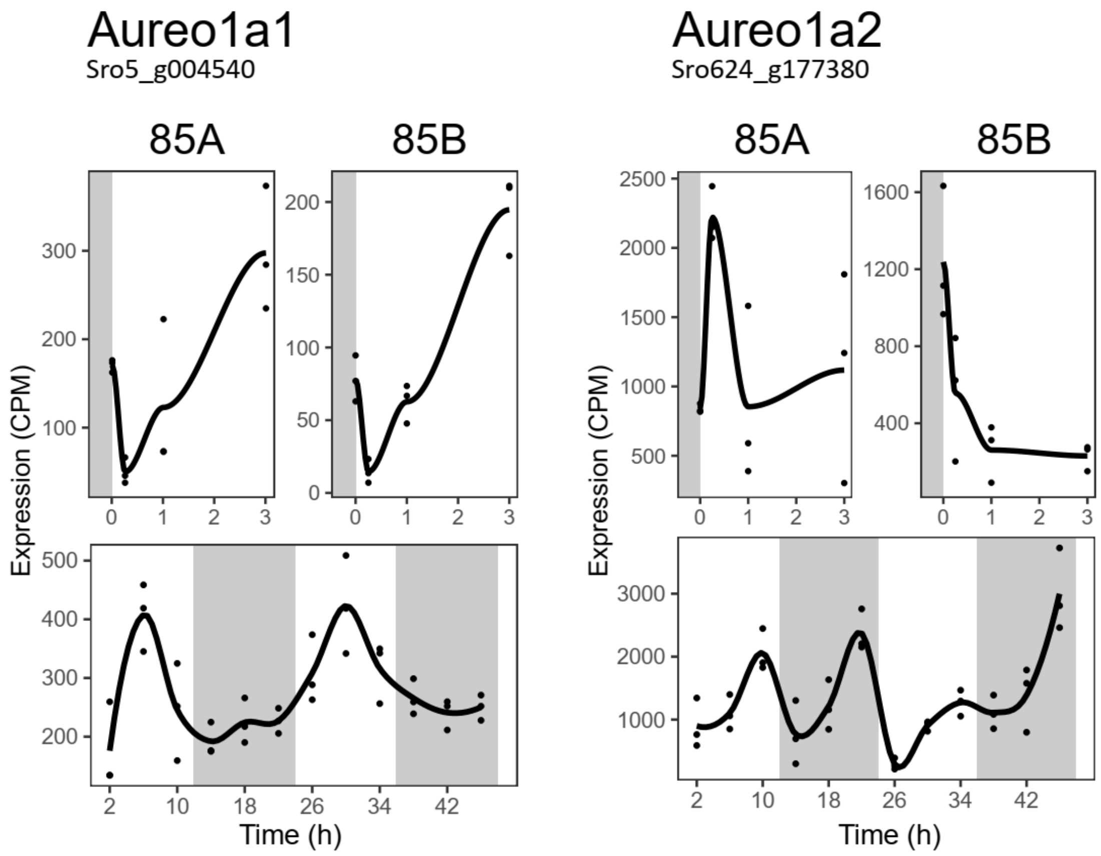

Figure S6: Expression over time (in h) in counts per million (CPM) of the two *S. robusta* Aureo1a homologs. On top: transcriptome data representing the response to light after a prolonged dark arrest from Moeys et al. (2016) [1] (strain 85B) and Bilcke et al. (2021a) [2] (strain 85A). Below: expression throughout a 2-day time series in a 12/12 day/night rhythm retrieved from Bilcke et al. (2021b) [3].

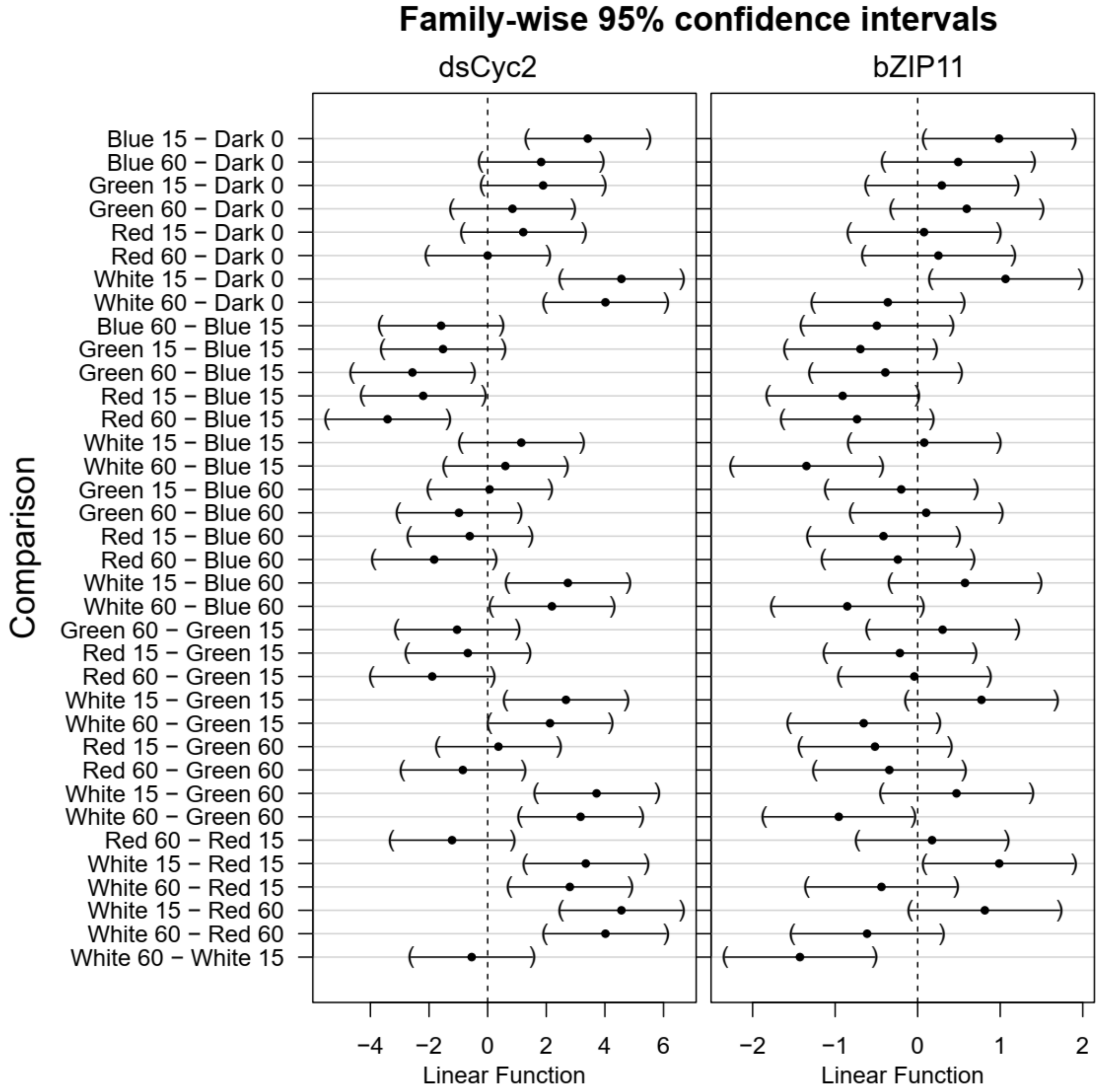

Figure S7: adjusted 95% confidence intervals for Tukey's pairwise comparisons testing for a difference in relative expression (log fold change versus the reference treatment) between different treatments. Treatments are shown on the y-axis and consist of different spectra as well as time since illumination (in minutes). RT-qPCR was performed for two genes, dsCyc2 (left) and bZIP11 (right).

Table S1: correction factors and light intensities (photon flux density, PFD) used for correction of each filter in spectral experiments. The final column contains the PFD (in µE m^{-2} s^{-1}) received by cultures covered with a color filter. The wavelength of maximum transmittance (*T*), the maximum transmittance and the correction factor to compensate for the transmittance are also presented.

Filter	Arg max $T(\lambda)$ (nm)	T_{max} (%)	Correction factor	Intensity applied (µE m^{-2} s^{-1})	Intensity received (µE m^{-2} s^{-1})
Full spectrum ("white")	/	100	1.00	61	61
#183 Moonlight Blue	455	71.52	1.39	85	19.1
#363 Special Medium Blue	447	49.21	2.02	123	10.4
#139 Primary Green	525	34.93	2.85	174	15.1
#787 Marius Red	709	84.51	1.18	72	6.6
#789 Blood red	709	43.71	2.28	139	7.7

Table S2: identification of homologs of candidate *P. tricornutum* light-dependent cell cycle checkpoint genes in the *S. robusta* reference genome using the PLAZA Diatoms integrative orthology framework. *S. robusta* homologs are scored in three categories: best-hits-and-inparalogs family (BHIF), phylogenetic tree-based homology (Tree-based), and belonging to the same orthologous gene family (Gene family). The relationship in terms of number of orthologs in each species is shown in the final column, with the first number indicating the number of homologs in *P. tricornutum* and the second the number of corresponding orthologs in *S. robusta* based on this analysis.

Gene name	*P. tricornutum* gene ID	*S. robusta* gene ID	BHIF	Tree-based	Gene family	Orthology relationship
dsCyc2	ptri158990	Sro756_g197840	yes	yes	yes	One-one
bZIP11A	ptri39200	Sro980_g227420	yes	yes	yes	One-two
bZIP11B	ptri39200	Sro589_g171710	yes	yes	yes	One-two
Aureo1c	ptri135580	Sro971_g226430	yes	yes	yes	One-one
CPF1	ptri139530	Sro82_g043740	yes	yes	yes	One-one
bZIP10	ptri210280	Sro23_g015840	yes	yes	yes	One-one
Aureo1a1	ptri48450	Sro5_g004540	yes	yes	yes	One-two
Aureo1a2	ptri48450	Sro624_g177380	no	yes	yes	One-two

Table S3: Results of differential expression (DE) analysis for *S. robusta* homologs of light-dependent cell cycle checkpoint genes from *P. tricornutum*. DE between 15 minutes of light and darkness was assessed using the RNA-seq data from Bilcke et al. (2021a) [2] and Moeys et al. (2016) [1]. Log_2 fold changes of light versus dark for each data set are indicated as "logFC 85A" and "logFC 85B". FDR adjusted p-values are given for the separate hypotheses (AdjP 85A, AdjP 85B) and are aggregated over both data sets ("Sidak min", tests for DE in at least one mating type). Finally, "diurnal phase" indicates the phase (timing of maximum expression, in hours since first light onset) measured in the 12/12 light/dark RNA-seq experiment of Bilcke et al. (2021b) [3].

		**Log2 fold change (light vs dark)**		**Adjusted p-values**		**Adjusted aggregated p-values**	**Diurnal phase**
gene name	gene_ID	logFC 85A	logFC 85B	AdjP 85A	AdjP 85B	**Sidak min** DE in ≥ 1 Strain	phase (h)
dsCyc2	Sro756_g197840	3.24	3.86	**0.000165**	**0.000289**	**0.000238**	Phase 2
bZIP11A	Sro980_g227420	1.91	1.15	**0.001237**	**0.095906**	**0.001988**	NA
Aureo1c	Sro971_g226430	2.17	3.12	**0.000986**	**0.000004**	**0.000003**	Phase 2
CPF1	Sro82_g043740	2.15	2.39	**0.000762**	**0.005916**	**0.001216**	Phase 2
bZIP10	Sro23_g015840	-0.14	0.76	0.850385	0.310016	0.362053	NA
bZIP11B	Sro589_g171710	-1.41	-0.20	**0.015044**	0.861714	**0.024642**	NA
Aureo1a1	Sro5_g004540	-1.49	-2.05	**0.010360**	**0.002740**	**0.002320**	Phase 6
Aureo1a2	Sro624_g177380	1.69	-0.77	**0.006368**	0.334755	**0.010399**	Phase 22

References

[1] Moeys S, Frenkel J, Lembke C, Gillard JTF, Devos V, Van Den Berge K, et al. A sex-inducing pheromone triggers cell cycle arrest and mate attraction in the diatom *Seminavis robusta*. Sci Rep 2016;6:19252.

[2] Bilcke G, Van den Berge K, De Decker S, Bonneure E, Poulsen N, Bulankova P, et al. Mating type specific transcriptomic response to sex inducing pheromone in the pennate diatom *Seminavis robusta*. ISME J 2021;15:562–76.

[3] Bilcke G, Osuna-Cruz CM, Silva MS, Poulsen N, D'hondt S, Bulankova P, et al. Diurnal transcript profiling of the diatom *Seminavis robusta* reveals adaptations to a benthic lifestyle. Plant J 2021:tpj.15291.
